# Compliance to the Alcohol Law: Overserving to Obviously Intoxicated Visitors at Music Festivals

**DOI:** 10.3390/ijerph17228699

**Published:** 2020-11-23

**Authors:** Kristin Feltmann, Johanna Gripenberg, Tobias H. Elgán

**Affiliations:** STAD, Centre for Psychiatry Research, Department of Clinical Neuroscience, Karolinska Institutet & Stockholm Health Care Services, Region Stockholm, 113 64 Stockholm, Sweden; kristin.feltmann@ki.se (K.F.); johanna.gripenberg@ki.se (J.G.)

**Keywords:** pseudo-intoxication, pseudo-patron, alcohol policy, alcohol prevention, large events

## Abstract

Music festivals are often high-risk settings associated with large numbers of visitors and high alcohol intoxication levels, which contribute to a number of public health-related problems. According to the Swedish Alcohol Act, servers are responsible for not overserving alcohol to obviously intoxicated patrons. The aim of the current study was to examine compliance to the Swedish Alcohol Act at music festivals by assessing the rate of alcohol overserving to festival-goers. We conducted a study at a large music festival in Sweden hosting approximately 50,000 visitors. Professional actors, i.e., pseudo-patrons, enacted a standardized scene in which a highly intoxicated festival-goer attempted to buy beer at licensed premises inside the festival. Observers monitored each attempt. A total of 52 purchase attempts were conducted. The rate of overserving was 26.9% and was not influenced by the server’s gender, the number of servers, or the level of crowdedness at the bar area. Overserving differed between server age groups, which was not statistically significant when controlling for other factors. Compliance to the Alcohol Act at the festival can be improved. Intoxication levels and related problems can be reduced by implementing a multicomponent intervention including staff training, policy work, and improved enforcement.

## 1. Introduction

Music festivals are events where several thousands of people, mainly young, gather and socialize, often over several days, and alcohol is usually a focal point that contributes to the festival experience. High levels of alcohol intoxication among festival-goers [1,2,3] and risky consumption [4,5,6] are concerning, since alcohol increases the risk for a number of public health-related problems such as injuries, sexual harassment, and violence [7,8,9], especially in large crowds [10].

In Sweden, a license issued by the local Alcohol Licensing Board is a requirement for selling alcoholic beverages at establishments [11]. Serving alcohol to adolescents under 18 years of age as well as to adults who are obviously intoxicated (i.e., overserved) is prohibited [12]. Servers are personally responsible for complying with these rules and risk fines or up to 6 months in prison for noncompliance [13]. Decreasing the level of overserving is a means to decrease intoxication levels. To test the level of overserving, previous studies have used professional actors, i.e., pseudo-patrons, portraying a standardized scene of obvious intoxication while attempting to purchase alcohol at licensed premises [14,15,16,17,18,19], sports stadiums [20,21], or community festivals [22]. For example, in a study conducted in Sweden, overserving was observed in 95% of the attempts [16]. Subsequent implementation of an environmental, community-based, multicomponent “Responsible Beverage Service” (RBS) intervention decreased the rates of overserving to 53% and 30% at the 3- and 5-year follow-up, respectively [16]. Similar effects have been demonstrated in the United Kingdom and the United States [17,19], and a systematic review concluded that RBS training, policy work, and improved enforcement may be effective in reducing assaults, traffic accidents, and underage sales [23]. A recent study also demonstrated that a community-based intervention implemented in Spain reduced underaged alcohol sales from 77% at baseline to 46% at the 2-year follow-up [24].

To examine compliance to the Swedish Alcohol Act at music festivals, we assessed the rate of alcohol overserving to festival-goers at the same festival at which we recently demonstrated high levels of alcohol intoxication among festival-goers [1].

## 2. Materials and Methods

The rate of overserving was tested at all bars (*n* = 12) during days two and three of a large four-day outdoor music festival (approximately 50,000 visitors) in Sweden in the summer of 2017. The purchase attempts were conducted from 7:00 p.m. to the bar closing time at 1:00 a.m. Alcohol could not be brought into the festival area, but could be purchased and consumed inside the entire festival area. Purchases were made using a cash-loaded chip attached to a bracelet. Approximately 40 “alcohol inspectors,” whose task was to help or, if necessary, dismiss intoxicated individuals, were hired by the organizers. There were two larger bars (>10 counters), five medium-sized bars (3–10 counters), and five smaller bars (<3 counters). Adjacent to the festival area were eight camping sites where the majority of the festival-goers resided (the largest camping site had room for up to 20,000 campers). Alcohol could be brought to the camping sites, and preloading was common at these sites.

Two male professional actors, visibly appearing of legal drinking age, were trained to perform a standardized scene of obvious alcohol intoxication as judged by an expert panel [16]. When entering the bar area, several signs of obvious intoxication were acted out including slurred speech, staggering, and having problems focusing and fixing the gaze. Once the actor had the attention of the server, the actor ordered a beer while portraying these signs of intoxication. If requested to pay, the actors first tried to pay in a clumsy way using the arm not holding the cash-loaded bracelet. This procedure assured that the actors portrayed a level of intoxication so that there was no doubt the actors were too intoxicated to be served alcohol. Before each attempt, a research assistant stood in close proximity to the bar so that he or she had a good overview of the bar area and the serving staff. The research assistant then observed the serving staff as the actor approached the bar. After each attempt, the research assistant together with the actor filled out a digital protocol using a mobile phone. The following factors were recorded: the time of the purchase attempt, ambience of the bar area (“calm” to “very busy” on a 5-point Likert scale), and the server’s estimated age, gender, and reaction prior to and during the purchase attempts (e.g., the level to which the server paid attention to the intoxicated visitor from “not at all” to “very clearly” on a 5-point Likert scale so that, for instance, if the server reacted to the actor by hesitating to proceed with the purchase or initiating an conversation with the actor other than taking the order, it was assessed as “very clearly”). At the large and medium-sized bars, the same counter was never tested more than once throughout the entire study. The festival organizers were not informed about these purchase attempts. The study was approved by the Regional Ethical Review Board in Stockholm (no. 2011/343-31).

Results are presented in frequencies and analyzed by Pearson’s chi-square or Fisher’s exact tests. Multivariate logistic regression was used to analyze factors associated with rates of overserving. The following factors were included: server’s gender, server’s age (categorized in 18–25 and >25 years old), bar type (small, medium, and large sized), and time of purchase attempt (categorized as 7:00 p.m.–8:59 p.m., 9:00 p.m.–10:59 p.m., and 11:00 p.m.–12:59 a.m.).

## 3. Results

In total, 52 purchase attempts of beer were conducted at the 12 bars by the two pseudo-intoxicated actors. In 26.9% (*n* = 14) of these attempts, alcohol was served. The bar area was not busy in the majority of attempts (in 87%, *n* = 45, of the attempts, the bar area was rated as “calm,” i.e., options “1” and “2” on the 5-point Likert scale). In 61% of the attempts (*n* = 31), the servers did not pay any particular attention to the actor’s intoxication level before the purchase attempt (i.e., the attention was rated as a “1,” “2,” or “3” on the 5-point Likert scale). However, in 39% (*n* = 20) of the attempts, the serving staff did pay attention to the intoxication level of the actors (i.e., the attention was rated as a “4” or “5” on the 5-point Likert scale). During the purchase attempts, almost all servers paid attention to the intoxication level of the actors, as the observers rated the attention level of the servers as a “4” (10%, *n* = 5) or “5” (89%, *n* = 46) on the 5-point Likert scale.

When denying alcohol service to the actors (*n* = 38), in almost all cases (97%, *n* = 37), the actors were told it was because they were deemed as being too intoxicated. In 74% (*n* = 28) of these cases where alcohol service was denied, the servers recommended alcohol-free alternatives. A colleague was asked for advice in 13% (*n* = 5) of the cases when alcohol was denied. In 5% (*n* = 2) of the cases the actor was asked to wait with their order, and in one case the actor was asked to leave the bar area. When alcohol was denied, the server never ignored the actor, called security or inspectors, nor encouraged the actor to buy an alcoholic drink. 

Rate of overserving did not differ between the actors or the genders of the servers, but differed between age groups of the servers and the time of purchase attempt (Table 1). A multivariate logistic regression analysis was conducted using the outcome of the purchase attempts as the dependent variable and the following independent variables: age group of the server, server’s gender, bar type, and the time of purchase attempt. Results showed that the time of purchase attempt remained statistically significant as actors were more likely to be served early in the evening (Table 2).

## 4. Discussion

Actors portraying signs of obvious alcohol intoxication were served alcohol at a large music festival in more than one of four attempts, indicating that an intoxicated person can easily obtain alcohol after a few attempts at different bars. Thus, there is room for improvement with regards to compliance to the Alcohol Act, which states that alcohol is not to be served to obviously intoxicated patrons. We have previously shown that every third visitor at the same festival was heavily intoxicated [1], similar to a festival in Norway [2]. Overserving at licensed premises at the festival is one factor that contributes to the overall high intoxication level among visitors. To prevent high levels of alcohol intoxication and related problems, such as sexual harassment and violence, and to comply with the Alcohol Act, obviously intoxicated festival-goers should be denied service of alcohol and also be denied entrance to the festival area since “preloading” in adjacent camping sites is a common phenomenon. Yet another strategy would be to ban alcohol service and consumption at the festival. In fact, “alcohol-free” festivals have taken place in Sweden. 

In the current study, the rate of overserving (26.9%) was similar to those measured at bars in Stockholm [16,20] but significantly lower than those observed at licensed premises in other countries (82–86%) [14,15,18] and at sports stadiums (74–75%) [20,21]. These differences could be due to potential methodological or circumstantial differences across these studies. Another explanation is the wide dissemination and implementation of the environmental multicomponent RBS program at bars in Stockholm [16]. This program consists of community mobilization and collaboration, RBS training, and improved enforcement and policy work, and it has shown to decrease the level of overserving [16], service to underaged [25], and to decrease alcohol-related violence by 29% [26], rendering a cost–benefit ratio of 1:39 [27]. Thus, tailoring and implementing an RBS intervention for the music festival setting holds promise to be beneficial. One challenge specific to the music festival setting is the large number of extra staff working during the event, which may be a difficult group to reach with regular face-to-face training. Providing RBS training online is a feasible way to provide training and has previously shown positive effects [28].

In the present study, the likelihood of being served did not differ between the two actors. Nor did server’s gender or age affect the likelihood of being served, similar to previous studies [14,15,16,20]. The only factor that emerged as influencing overserving was the time when the purchase attempt was conducted. It was shown that the likelihood for the actors to be overserved was greater earlier in the evening. Reasons may be that there were different staff working earlier in the evenings, as opposed to later, and that the focus among serving staff on alcohol and intoxication was possibly not as common early in the evenings. In addition, the aforementioned alcohol inspectors were perhaps, to a higher degree, present at the bars later in the evenings. In our previous study of overserving at licensed premises, the extent to which the server noticed the intoxication level before and during the attempts influenced the likelihood of being served [20]. Furthermore, previous studies show that the level of crowdedness can influence the likelihood of overserving [14,15]. The fact that the bar areas were rather calm during the vast majority of attempts might have contributed to the relatively low rate of overserving, observed in the current study. 

A limitation of this study was that only male actors were used. Although one study showed that female pseudo-buyers were served to a higher extent than male counterparts [14], another study demonstrated contrary results [15]. It is, therefore, possible that results would have been different if we had used female pseudo-buyers, although it is unclear in what direction. Further, as we only conducted the study at one festival, the generalizability of the findings is limited. Finally, the sample size can be considered quite small. However, we believe that the sample generates results sufficient to pin-point areas of improvement so festivals can comply to the Alcohol Act. 

## 5. Conclusions

Alcohol is often a focal point at music festivals, and high intoxication levels among visitors are problematic. According to the Swedish Alcohol Act, obviously intoxicated individuals should not be served alcohol. The present findings demonstrate that compliance to the Alcohol Act at music festivals can be improved by decreasing the rates of overserving. Research has shown that the rate of overserving can be reduced by the development and implementation of environmental multicomponent intervention strategies including, for instance, staff RBS training, alcohol policy work, and improved enforcement by authorities. We, therefore, suggest that such prevention strategies should be developed and tested at music festivals. 

## Figures and Tables

**Table 1:** Rates of overserving across various characteristics.

Characteristic	Proportion of Purchase Attempts % (*n*)	Proportion of Overserving % (*n*)	x^2^(df) ^a^, *p*
Actor identity Actor 1 Actor 2	52 (27) 48 (25)	15 (4) 40 (10)	*p* = 0.061 ^b^
Server’s gender Female Male	58 (30) 42 (22)	27 (8) 27 (6)	0.002 (1), *p* = 0.961
Server’s age 18–25 years 26–35 years >35 years	35 (18) 60 (31) 6 (3)	11 (2) 39 (12) 0 (0)	*p* = 0.050 ^b^
Time of purchase 7:00–8:59 p.m. 9:00–10:59 p.m. 11:00 p.m.–12:59 a.m.	23 (12) 39 (20) 39 (20)	50 (6) 30 (6) 10 (2)	*p* = 0.044 ^b^
Bar type ^c^ Small sized Medium sized Large sized	17 (9) 48 (25) 35 (18)	33 (3) 28 (7) 22 (4)	*p* = 0.844 ^b^

^a^ df = degrees of freedom, ^b^ Fisher’s exact test since at least one cell had an expected count <5. ^c^ Small-sized bars had <3 counters, medium-sized bars had 3–10 counters, and large-sized bars had >10 counters.

**Table 2:** Logistic regression analysis of factors influencing overserving.

Independent Variables	Odds Ratio	95% CI ^a^	*p*
Server’s gender (ref: male)			
Female	0.82	0.17–3.89	0.806
Server’s age (ref: 18–25 years old)			
>25 years old	3.46	0.56–21.33	0.181
Bar type (ref: small sized) ^b^			
Medium sized	0.57	0.08–4.13	0.575
Large sized	0.58	0.07–4.60	0.609
Time of purchase (ref: 7:00–8:59 p.m.)			
9:00–10:59 p.m.	0.37	0.07–2.04	0.252
11:00 p.m.–12:59 a.m.	0.10	0.01–0.77	0.027

Nagelkerke R^2^ = 25.6%, ^a^ CI—confidence interval. ^b^ Small-sized bars had <3 counters, medium-sized bars had 3–10 counters, and large-sized bars had >10 counters.
